# Hyperpolarized [2–^13^C]pyruvate MR molecular imaging with whole brain coverage

**DOI:** 10.1016/j.neuroimage.2023.120350

**Published:** 2023-08-25

**Authors:** Brian T. Chung, Yaewon Kim, Jeremy W. Gordon, Hsin-Yu Chen, Adam W. Autry, Philip M. Lee, Jasmine Y. Hu, Chou T. Tan, Chris Suszczynski, Susan M. Chang, Javier E. Villanueva-Meyer, Robert A. Bok, Peder E.Z. Larson, Duan Xu, Yan Li, Daniel B. Vigneron

**Affiliations:** a Department of Radiology and Biomedical Imaging, University of California, 1700 Fourth Street, Byers Hall Suite 102, San Francisco, CA 94158, USA; b UCSF – UC Berkeley Graduate Program in Bioengineering, University of California, USA; c ISOTEC Stable Isotope Division, MilliporeSigma, Merck KGaA, Miamisburg, OH 45342, USA; d Department of Neurological Surgery, University of California, San Francisco, CA 94158, USA

**Keywords:** Hyperpolarized carbon-13, Molecular imaging, Brain Metabolism

## Abstract

Hyperpolarized (HP) ^13^C Magnetic Resonance Imaging (MRI) was applied for the first time to image and quantify the uptake and metabolism of [2–^13^C]pyruvate in the human brain to provide new metabolic information on cerebral energy metabolism. HP [2–^13^C]pyruvate was injected intravenously and imaged in 5 healthy human volunteer exams with whole brain coverage in a 1-minute acquisition using a specialized spectral-spatial multi-slice echoplanar imaging (EPI) pulse sequence to acquire ^13^C-labeled volumetric and dynamic images of [2–^13^C] pyruvate and downstream metabolites [5–^13^C]glutamate and [2–^13^C]lactate. Metabolic ratios and apparent conversion rates of pyruvate-to-lactate (*k*_PL_) and pyruvate-to-glutamate (*k*_PG_) were quantified to investigate simultaneously glycolytic and oxidative metabolism in a single injection.

## 1. Introduction

Hyperpolarized (HP) carbon-13 MR using dissolution Dynamic Nuclear Polarization (dDNP) has been investigated in animals since 2006 to provide a unique window into cellular metabolism, enabling the quantification of enzyme-catalyzed conversion rates that inform on critical cellular biochemistry in both normal and pathologic conditions as well as perfusion information (Ardenkjaer-Larsen et al., 2003; Golman et al., 2003; Golman et al., 2006; Kurhanewicz et al., 2019). A first-in-human proof-of-concept clinical trial of HP [1–^13^C]pyruvate completed in 2013 demonstrated feasibility and safety in patients (Nelson et al., 2013). The subsequent development of commercial research polarizers enabled new technical developments and initial human studies over the past 5 years in a variety of applications including prostate cancer, brain tumors, renal cancer, cardiac disease, pancreatic cancer, traumatic brain injury and breast cancer (Granlund et al., 2020; Miloushev et al., 2018; Cunningham et al., 2016; Abeyakoon et al., 2019; Gallagher et al., 2020; Stødkilde-Jørgensen et al., 2020; Tran et al., 2019; Aggarwal et al., 2017; Park et al., 2018; Autry et al., 2019; Grist et al., 2019; Autry et al., 2020; Gordon et al., 2020; Hackett et al., 2020; Autry et al., 2021; Kim et al., 2021; Li et al., 2021; Chen et al., 2021). Since 2018, studies have investigated cerebral energy metabolism with HP [1–^13^C]pyruvate in the normal brain and neuro-pathologies, demonstrating novel insights into brain bioenergetics by measuring the conversion of pyruvate to lactate catalyzed by the enzyme lactate dehydrogenase (LDH) and pyruvate to bicarbonate via pyruvate dehydrogenase (PDH) (Miloushev et al., 2018; Park et al., 2018; Autry et al., 2019; Grist et al., 2019; Autry et al., 2020; Gordon et al., 2020; Hackett et al., 2020; Autry et al., 2021; Kim et al., 2021; Li et al., 2021; Chen et al., 2021).

While HP [1–^13^C]pyruvate has successfully been utilized as a probe in over 500 patient studies to quantify the conversion to [1–^13^C]lactate and [^13^C]bicarbonate in the human brain, its metabolism and conversion to ^13^CO_2_ through PDH prevents direct detection of TCA cycle metabolism (Li et al., 2021). To address this, we investigated HP ^13^C-pyruvate labeled in the C2 position to provide a unique MR molecular imaging window into the TCA cycle as the labeled carbon is carried over to acetyl-CoA and enables the observation of [5–^13^C] glutamate after enzyme catalyzed conversion from α-ketoglutarate (Chung et al., 2019). In the human brain, glutamate is the most abundant free amino acid and is at a crossroad between multiple metabolic pathways (Petroff, 2002; Segovia et al., 2001). Glutamate plays a significant role as an excitatory neurotransmitter that is critical for neuronal signal transmission in the brain and throughout the nerves in the body and plays an important role during brain development while shaping learning and memory (Petroff, 2002). Glutamate has also been shown to decrease in aging and neurodegeneration, and *in vivo* MR spectroscopy studies exploring changes in neurometabolite concentrations in patients with grade 2 & 3 IDH-mutant gliomas detected reduced levels of glutamate (Segovia et al., 2001; Hoerst et al., 2010; Autry et al., 2022). Studies have also shown that conversion of α-ketoglutarate to glutamate is reduced in isocitrate dehydrogenase 1 (IDH1) mutant cells and brain tumor models providing additional information on IDH1 metabolic reprogramming (Chaumeil et al., 2014).

In order to investigate [2–^13^C]pyruvate metabolism in the brain, we developed the required methods for the production of sterile HP doses of GMP [2–^13^C]pyruvate (Chung et al., 2019). Our initial first-in-human study using non-localized HP ^13^C MR spectroscopy demonstrated the feasibility of obtaining human HP [2–^13^C]pyruvate brain data, but did not provide the ability to image metabolic conversions throughout the brain as is required for detection of both normal and pathologic variations (Chung et al., 2019). While [2–^13^C]pyruvate has the potential to provide direct information about TCA cycle metabolism, there are significant challenges in imaging its metabolic conversion due to its broad chemical shift dispersion, and lactate signals have a shorter T_1_ and peak-splitting due to J_CH_ coupling. Recent technical advances, including variable-resolution echo-planar imaging (EPI) (Gordon et al., 2020) and patch-based higher order singular value decomposition (HOSVD) (Kim et al., 2021), have enabled higher spatial resolution and improved image quality in HP ^13^C MRI. In this project, we developed a multi-slice, dynamic HP [2–^13^C]pyruvate EPI pulse sequence and incorporated spatiotemporal patch-based denoising methods to apply this technique for the first time in healthy volunteer studies with whole brain coverage to investigate its application for monitoring cerebral energy metabolism using HP [2–^13^C]pyruvate.

## 2. Material and methods

### 2.1. [2–^13^C]pyruvate preparation

Hyperpolarization was performed using a 5T SPINlab dDNP polarizer (GE Healthcare) operating at 0.8 K. Samples containing 1.47 g [2–^13^C] pyruvic acid (ISOTEC Stable Isotope Division, MilliporeSigma, Merck KGaA) and 15 mM electron paramagnetic agent (AH111501, GE Healthcare) were prepared the morning of the study and polarized for at least two hours. Samples were then rapidly dissolved using superheated water and the electron paramagnetic agent was removed by filtration prior to neutralization with a TRIS-buffered NaOH solution. Prior to injection, the pH, pyruvate and residual EPA concentrations, polarization, and sample temperature were rapidly measured with an integrated quality control (QC) module. In parallel, the integrity of the 0.2 μm sterile filter was tested in agreement with manufacturer specifications prior to injection. After release by the pharmacist, a 0.43 mL/kg dose of 241 ± 7 mM pyruvate (*n* = 5) was injected at a rate of 5 mL/s, followed by a 20 mL sterile saline flush (0.9% sodium chloride, Baxter Healthcare Corporation). The liquid-state polarization was 28.2 ± 6.8%, and the average time elapsed between the start of dissolution and the start of IV injection was 48.8 ± 1.9 s.

### 2.2. Human MR imaging protocol: [2–^13^C]pyruvate spectral-spatial pulse with EPI

**Fig. 1.**
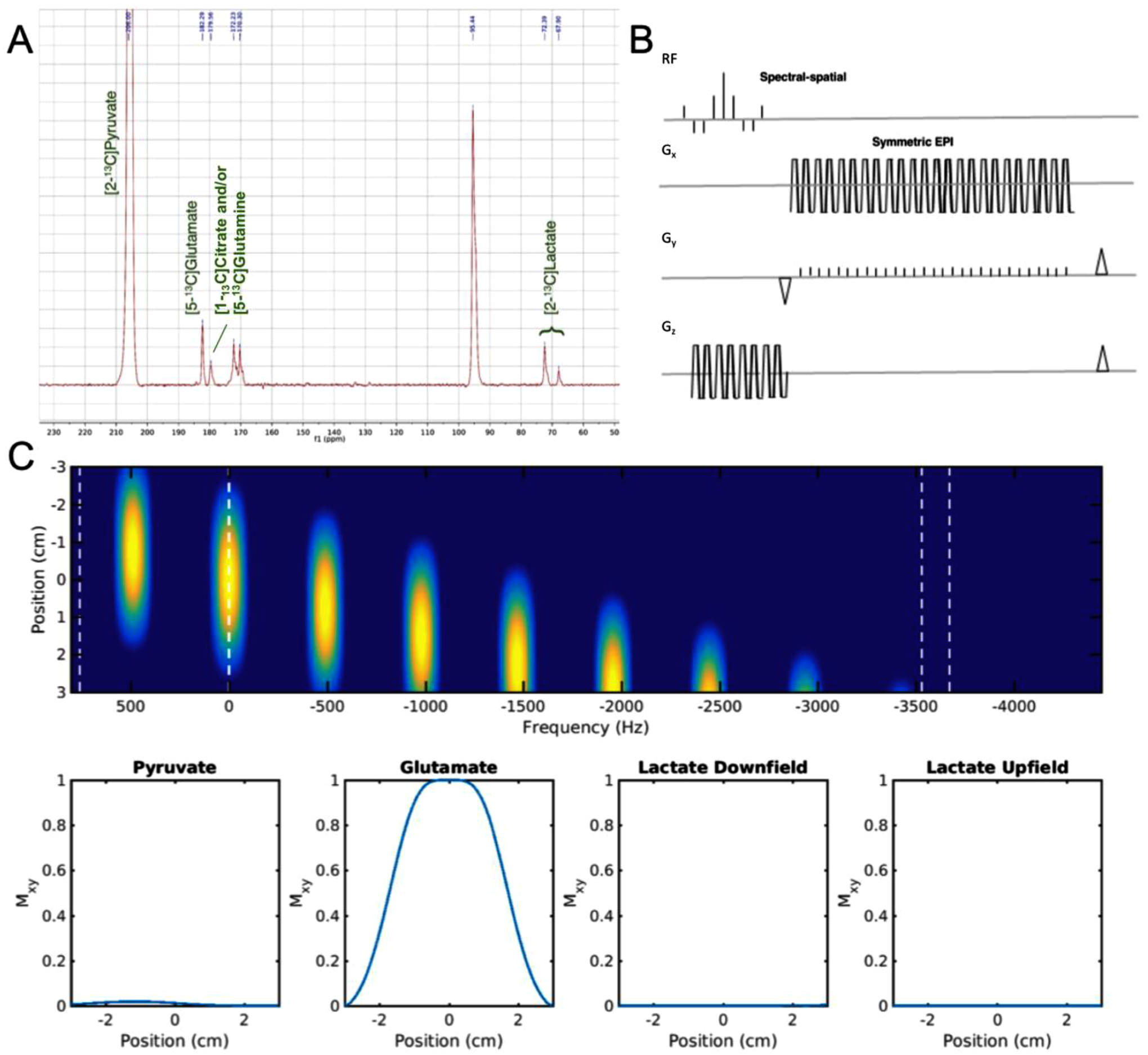
Singleband spectral-spatial RF pulse and EPI readout showing simulated responses from [2–^13^C]pyruvate, [5–^13^C]glutamate and [2–^13^C]lactate to demonstrate the effectiveness of designed pass & stopbands. A. Chemical shift frequencies of [2–^13^C]pyruvate and downstream metabolites were obtained from an NMR spectrum acquired in a previous study (Chung et al., 2019). The asymmetry of the lactate doublet is primarily due to the RF excitation band used. B. Sequence diagram illustrating the metabolite-selective EPI acquisition. C. The spectral-spatial response of the 2D RF pulse used to selectively excite [2–^13^C]pyruvate (206.00 ppm), [5–^13^C]glutamate (182.29 ppm), and the [2–^13^C]lactate doublet (72.39 ppm and 67.90 ppm) with minimal off-resonance excitation. The vertical dashed lines correspond to the relative frequency for each metabolite at 3T. See Supplementary Fig. 1 for frequency responses of the spectral-spatial RF pulse when centered on other resonances.

Imaging studies were performed on a 3T clinical MR system (MR750, GE Healthcare, Waukesha, WI) using a commercially available 8-channel ^1^H / 24-channel ^13^C head coil (RAPID Biomedical, Germany) with an integrated ^13^C birdcage volume exciter for RF transmit. The ^13^C RF power was calibrated using a head phantom containing natural abundance ethylene glycol prior to the study. All ^13^C MR data was acquired with a variable resolution, metabolite-selective EPI pulse sequence (Gordon et al., 2020). Using an RF toolbox (https://github.com/LarsonLab/hyperpolarized-mri-toolbox), the singleband spectral-spatial (SPSP) RF pulse was designed to independently excite [2–^13^C]pyruvate, [5–^13^C]glutamate, and downfield and upfield resonances of [2–^13^C]lactate with minimal off-resonance excitation (Fig. 1 and Supplementary Fig. 1). To avoid J-coupling artifacts, the downfield and upfield peaks of [2–^13^C]lactate were acquired independently. Using a multi-resolution approach, [2–^13^C]pyruvate was acquired with in-plane spatial resolution of 7.5 × 7.5 mm^2^ and 20° tip angle, while the downstream metabolites [5–^13^C]glutamate and the [2–^13^C]lactate doublet were acquired with an in-plane resolution of 22.5 × 22.5 mm^2^ and 60° tip angle. Five 3-cm slices were acquired with a matrix size of 32×32. 20 timeframes were acquired per metabolite with 3 s temporal resolution (TR) for a total scan time of 60 s. T_1_ -weighted ^1^H IR-SPGR (inversion recovery - spoiled gradient recalled) images and T_2_-weighted ^1^H FLAIR (fluid-attenuated inversion recovery) were acquired for anatomical reference. Four healthy volunteers were imaged following IRB and FDA-IND approved protocols with informed consent. Persons ranging in age from 29 to 60 years old were imaged at approximately midday with one volunteer scanned twice on different days yielding a total of 5 datasets.

### 2.3. Data analysis and quantitative post-processing

Following k-space noise pre-whitening, optimal coil combination techniques were performed across multichannel datasets with [2–^13^C] pyruvate end of scan timeframes serving as signalless floors to determine noise covariance (Kim et al., 2021; Zhu et al., 2019). Image reconstruction was performed using the Orchestra toolbox (GE Healthcare) in MATLAB. The HP ^13^C data was phased and denoised using a patch-based higher order singular value decomposition (HOSVD) method (Kim et al., 2021; Vaziri et al., 2022). In this study the following parameters were used in denoising all cases: kglobal = 0.4; klocal = 0.8 (scales used to determine thresholds); step = 2. In low-resolution cases: patch size = 3; radius of search window size = 4; and in high-resolution cases: patch size = 5; radius of search window size = 6. These parameters were chosen based on the optimal values found from the ^13^C MR EPI images of the human brain with HP [1–^13^C]pyruvate MRI (Kim et al., 2021). Signal maps for each metabolite were generated by normalizing voxels to global peak pyruvate signal. In the case of lactate, the upfield and downfield signals were individually processed and combined to yield a total lactate image. To assess image similarity between the upfield and downfield lactate images, structural similarity index measure (SSIM (Zhou et al., 2004)) was calculated using MATLAB. Area-Under-Curve (AUC) images for each metabolite were composed by summing data through time. From the AUC images, ratios of [5–^13^C]glutamate-to-[2–^13^C]pyruvate, [2–^13^C]lactate-to-[2–^13^C]pyruvate, and [5–^13^C]glutamate-to-[2–^13^C]lactate were calculated. Voxel-wise calculations for pyruvate-to-glutamate conversion rate (*k*_PG_) and pyruvate-to-lactate conversion rate (*k*_PL_) were performed using an irreversible, three-site exchange kinetic model (Larson et al., 2018). In the kinetic fitting, B_1_ field strength was scaled to 80%, and T_1_ relaxation time constants were chosen to optimally fit data. Analytical tools used for the kinetic fitting are available from the Hyperpolarized MRI Toolbox (https://github.com/LarsonLab/hyperpolarized-mri-toolbox). A brain mask for HP ^13^C data was created from *T*_1_- and *T*_2_-weighted images utilizing the FLIRT and FSL FAST algorithms for ^1^H image alignment and white matter segmentation (Jenkinson and Smith, 2001; Zhang et al., 2001). For kinetic analyses, a brain mask was created again with ^13^C brain voxels containing > 40% of white matter and gray matter and applied to the resulting AUC ratio and kinetic rate maps. When evaluating the average *k*_PL_, *k*_PG_, and AUC ratios for the whole brain, only the voxels with SNR of the AUC data (SNR_AUC_) greater than 3 for each metabolite were used, and those with fitting error less than 30% were used for calculating the average kinetic rates.

## 3. Results

### 3.1. Hyperpolarized [2–^13^C]pyruvate MR imaging

**Fig. 2.**
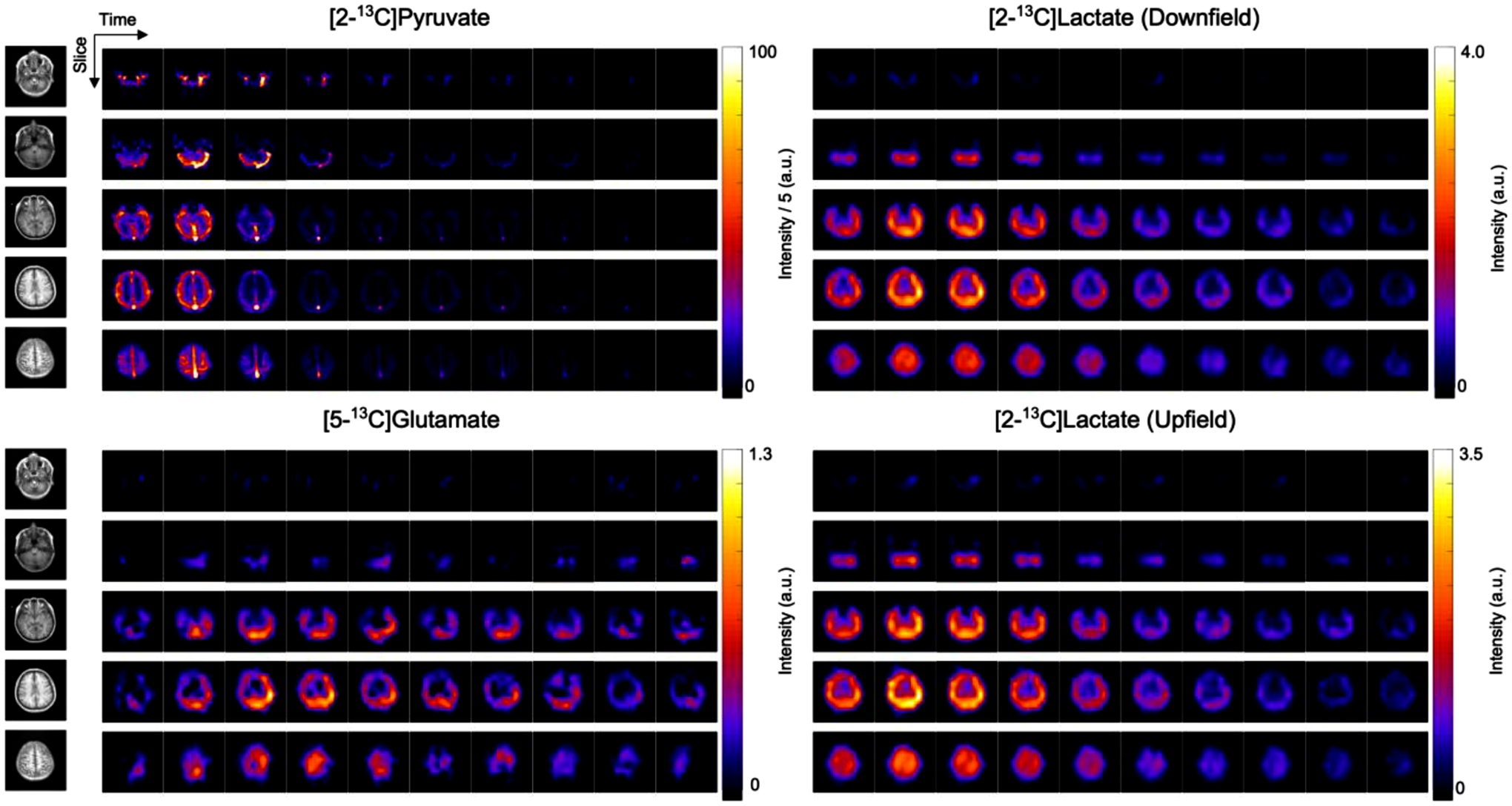
4D dynamics of HP [2–^13^C]pyruvate, [5–^13^C]glutamate, [2–^13^C]lactate (downfield peak) and [2–^13^C]lactate (upfield peak) from the human brain of a healthy volunteer (Vol-1). Displayed images show the first 10 timeframes with 3 second temporal resolution for a total of window of 30 s following denoising using a patch-based HOSVD method (Kim et al., 2021). Shown on the left are ^1^H IR-SPGR anatomy images capturing average of slices. Upper window levels for [2–^13^C]pyruvate data were adjusted to 20% of the maximum intensity. The images were denoised and zero-filled two-fold for display.

Fig. 2 shows denoised dynamic images of HP [2–^13^C]pyruvate, [5–^13^C]glutamate, and upfield and downfield [2–^13^C]lactate in the brain of a healthy volunteer. The dynamic upfield and downfield lactate images are free of ghosting or blurring that could have been attributed to J-coupling. Quantitatively, close agreement was observed between the upfield and downfield lactate images with high correlation (*r* = 0.97, Supplementary Figure 2) and a structural similarity index measure (SSIM (Zhou et al., 2004)) of 0.91 in this subject. Across all studies, *r* values of 0.85 ± 0.10 and SSIM of 0.88 ± 0.02 were obtained.

**Fig. 3.**
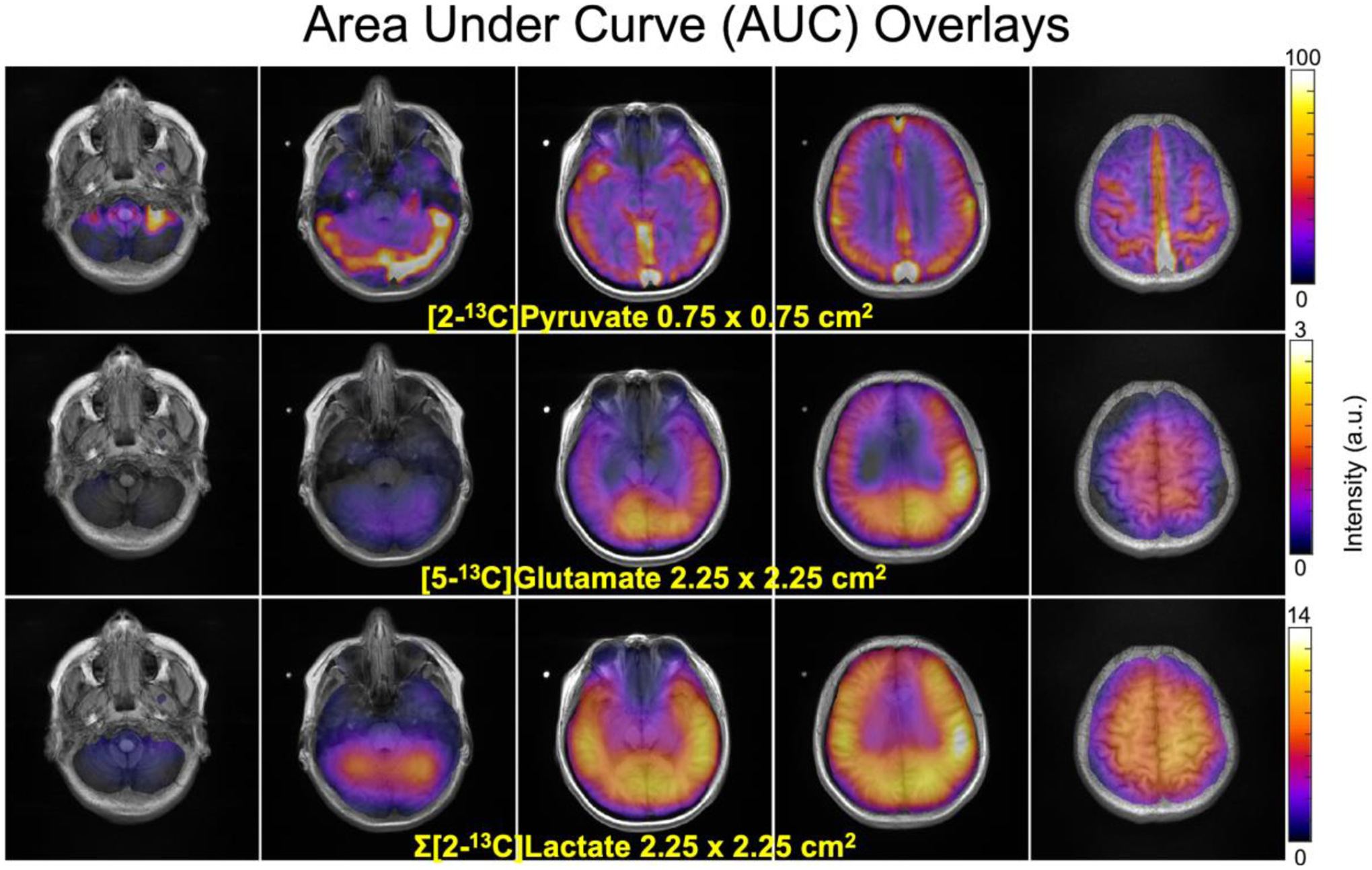
AUC (Area-Under-Curve) images from the volunteer (Vol-1) summed over 20 timeframes overlaid on ^1^H IR-SPGR images. [2–^13^C]lactate images illustrate acquired signal after summing both downfield and upfield peaks. High resolution (0.75 × 0.75 cm^2^) pyruvate data shows strong arterial and venous signal, and a brain mask to reduce the pyruvate signal from adjacent muscle was consequently applied. The images were denoised and zero-filled for display.

Fig. 3 shows AUC images from the same volunteer overlaid on ^1^H IR-SPGR images. Signal intensity of lactate and glutamate was normalized to the pyruvate signal. High resolution pyruvate data acquired at 0.75×0.75 cm^2^ shows strong arterial and venous signal well separated from surrounding tissue. Coarser resolution glutamate and lactate data acquired at 2.25×2.25 cm^2^ achieved sufficiently high SNR of whole brain coverage. With image denoising, the maximum SNR was 1147 for pyruvate, 65 for glutamate, and 278 and 275 for downfield and upfield lactate signals, respectively, which were 5.6-fold higher for pyruvate, 3.7 for glutamate, and 5.5 for downfield and 6.5 for upfield lactate relative to the raw SNR in this volunteer. The comparison of pre- and post-denoised images are displayed in Supplementary Figure 3. The mean SNR gain from all 5 studies was 7.1 ± 1.0 for pyruvate and 4.5 ± 0.5 for glutamate, 5.4 ± 1.2 for downfield and 5.1 ± 1.0 for upfield lactate.

**Fig. 4.**
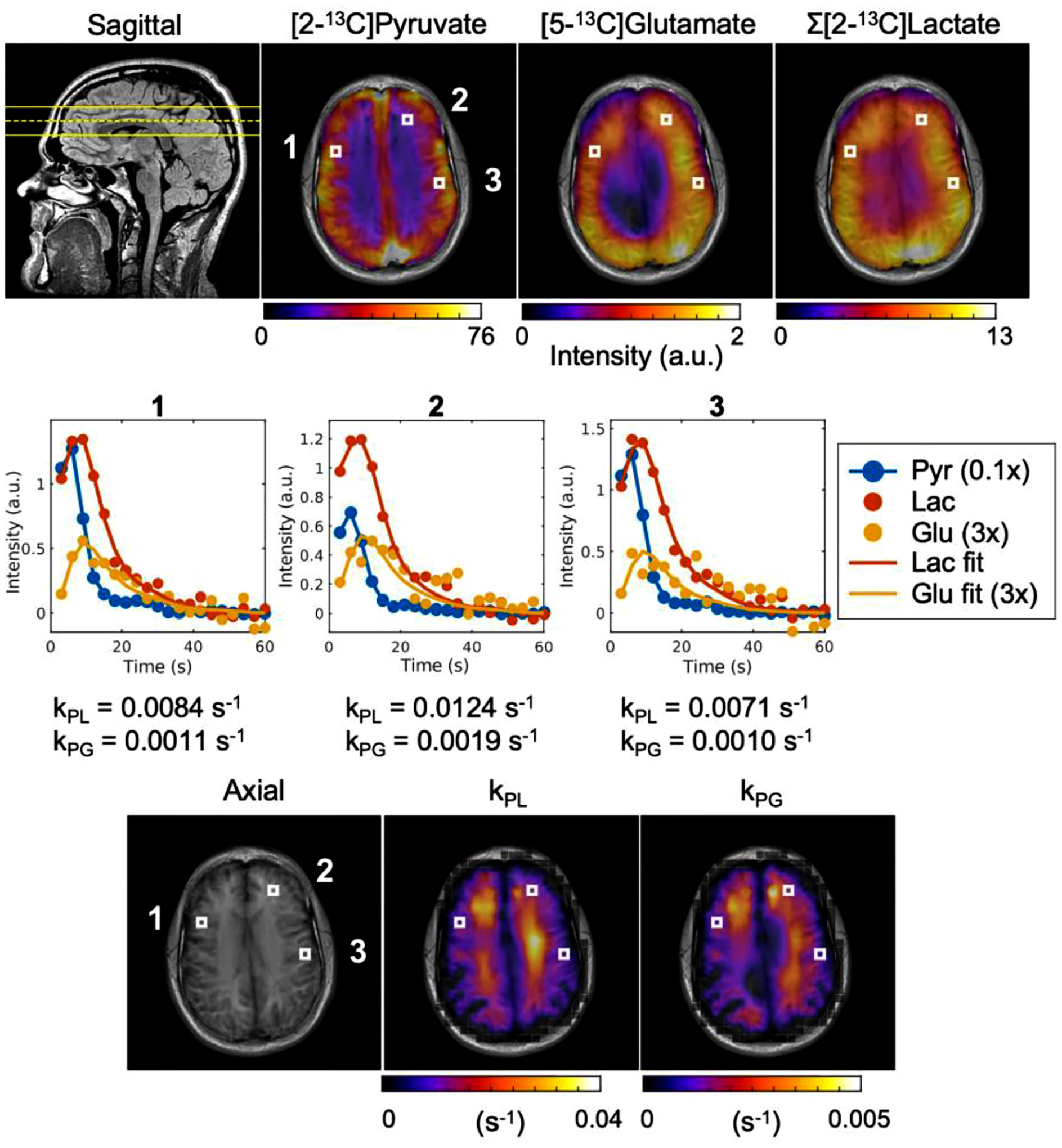
Kinetic analyses of dynamic HP [2–^13^C]pyruvate data. (Top row) A sagittal ^1^H FLAIR image from another volunteer showing a mid-slice position for ^13^C EPI data denoted with yellow lines (dashed/solid: center/edge of the slice) and denoised ^13^C AUC images for pyruvate, glutamate, and lactate overlaid on a corresponding axial ^1^H SPGR image. (Middle row) Signal intensities of pyruvate, glutamate, and lactate over time from the selected voxels indicated in the AUC images. Kinetic fits for lactate and glutamate are displayed. (Bottom row) Calculated *k*_PL_ and *k*_PG_ maps overlaid on axial ^1^H SPGR image were obtained from healthy volunteer brain data. The selected voxels are also indicated in the *k*_PL_ and *k*_PG_ maps.

Top row images in Fig. 4 show ^13^C AUC images of pyruvate, glutamate, and summed lactate (downfield and upfield) from a separate volunteer. The sagittal image depicts the anatomical position calculated from the DICOM header of the acquired slice. Adjacent signal overlays show similar distributions of [2–^13^C]pyruvate and downstream metabolites of [2–^13^C]lactate and [5–^13^C]glutamate as those observed in the volunteer data above (Vol-1). Also, both lactate and glutamate signals are high in cerebral cortex but low in white matter while signals in the left posterior of the brain can be seen higher than the contralateral side.

### 3.2. Kinetic rates (k_PG_, k_PL_) and metabolite AUC ratios

From the dynamic images of pyruvate, lactate, and glutamate signals, kinetic rates and metabolite AUC ratios can be determined. For example, middle-row plots in Fig. 4 present traces of pyruvate and its metabolite signals from selected voxels in three disparate regions and the kinetic fits determined for lactate and glutamate. Each selected voxel dynamics in the middle row showed *k*_PL_ values of 0.0084, 0.012, and 0.0071 s^−1^ and *k*_PG_ values of 0.0011, 0.0019, and 0.0010 s^−1^, respectively. The representative *k*_PL_ and *k*_PG_ maps obtained from healthy volunteer brain data are shown overlaid on the relevant axial T_1_-weighted image (bottom row images in Fig. 4). The average of voxelwise *k*_PL_ and *k*_PG_ values from five datasets were determined to be 0.0096 ± 0.0008 s^−1^ and 0.0014 ± 0.0001 s^−1^ which was a magnitude of order lower than *k*_PL_. The number of voxels comprising the *k*_PL_ maps increased by 1.2 to 3.1 times with denoising as more voxels with *k*_PL_ fulfilled the SNR and fitting error criteria, resulting in covering 89 to 99% of the brain voxels. Likewise, the *k*_PG_ coverage was increased by 2 to 7 times after denoising. AUC ratios of Σ[2–^13^C]lactate-to-[2–^13^C]pyruvate, [5–^13^C]glutamate-to-[2–^13^C]pyruvate, and [5–^13^C]glutamate-to-Σ[2–^13^C]lactate were calculated voxel-by-voxel from the five volunteer datasets, and the mean (± S.E.) values were 0.26 ± 0.02, 0.037 ± 0.004, and 0.14 ± 0.01, respectively.

## 4. Discussion

In this project, multi-slice, dynamic HP [2–^13^C]pyruvate EPI was developed and applied in healthy volunteer studies for the first time to investigate its in-vivo application for human brain research. A variable resolution multi-slice EPI approach to achieve whole brain coverage was utilized enabling the acquisition of glutamate images and a quantification of spatial and temporal distribution for future studies and pathology (Gordon et al., 2020). The observed spatial distributions of [2–^13^C] lactate and [5–^13^C]glutamate were consistent with previous studies on the lactate and bicarbonate (indirect measure of a flux to oxidative metabolism) distributions from HP [1–^13^C]pyruvate MRI (Lee et al., 2020) and the glutamate map from steady-state ^1^H MRSI (Bogner et al., 2012; Hangel et al., 2021) in the normal human brain. However, in brain tumors, it is expected that [2–^13^C]lactate signal would be high and ^13^C-glutamate signal would be low in lesions due to reduced oxidative energy metabolism, similar to the results from a [1–^13^C]pyruvate brain tumor study that demonstrated the lactate signal was elevated in the tumors while a pyruvate-to-bicarbonate conversion rate (*k*_PB_) was greatly reduced compared to normative brain (Park et al., 2018). We observed a slight asymmetry of metabolite signals in the left and right hemisphere, which may be caused by inhomogeneous coil sensitivity or perhaps there is actually an asymmetry in the metabolites. To correct for RF inhomogeneity, applying an intensity correction method such as using ^23^Na sensitivity profile (Sanchez-Heredia et al., 2022), a numerical model with known coil location and dimensions (Dominguez-Viqueira et al., 2016), and bias correction method (Lu et al., 2022) can be considered.

Due to a lower ^13^C polarization and faster *T*_1_ relaxation of [2–^13^C] pyruvate (*T*_1_ = 47 s (Chung et al., 2019)), we used a coarser spatial resolution for lactate and glutamate than those typically used in [1–^13^C] pyruvate MRI studies of the human brain. A patch-based higher-order singular value decomposition (HOSVD) denoising method was applied to datasets to further improved visualization of the metabolite signals and quantification of *k*_PL_ and *k*_PG_ kinetic conversion rates (Kim et al., 2021). With denoising, the number of voxels with *k*_PL_ fulfilling the SNR and fitting error criteria increased by approximately 3-fold, resulting in 89 to 99% brain coverage, and the *k*_PG_ coverage was increased by 2- to 7-fold depending on the SNR of the original data. While low SNR in the raw data can bias quantification, error in the estimated rate constants is expected to be less than 20% when the original SNR_AUC_ ≥ 3 (Kim et al., 2021). Other methods to improve image quality, including deuteration of the dissolution media as well as substrate (Keshari and Wilson, 2014) could preserve polarization and help improve SNR and consequently spatial resolution to better characterize cerebral metabolism.

We observed an average *k*_PG_-to-*k*_PL_ ratio of ~1/7, which is smaller than the ~1/3 to ~1/6 *k*_PB_-to-*k*_PL_ ratio observed with HP [1–^13^C]pyruvate (Kim et al., 2021; Grist et al., 2019). These relative values are in agreement with the underlying biochemistry, as conversion of pyruvate-to-bicarbonate by PDH occurs before conversion to glutamate as a byproduct of the TCA cycle and are both quantified by a single unidirectional rate constant. A unidirectional kinetic model was used to determine the kinetic rates since it has been shown to be a simple and robust approach to quantify metabolic conversion of hyperpolarized substrates (Larson et al., 2018; Sun et al., 2018; Mammoli et al., 2020). However, recent studies using HP [1–^13^C]-pyruvate MRI study have provided further evidence that astrocyte-neuron lactate shuttling contributes to the [1–^13^C]lactate and ^13^C-bicarbonate metabolism in the human brain (Brooks, 2018; Uthayakumar et al., 2023). This suggests that a more realistic model, which considers compartmentalization and the reverse reaction of lactate-to-pyruvate, may be needed to recapitulate the underlying neurometabolism and will be explored in future work.

Due to J_CH_-coupling, the [2–^13^C]lactate signal appears as a doublet with a coupling constant of 146 Hz (Marjańska et al., 2010). This peak splitting needs to be accounted for in the acquisition to avoid deleterious off-resonance artifacts and signal cancelation. As shown by Datta and Spielman (2021), one approach could be to simultaneously excite both peaks and acquire data at two different echoes separated with a spacing of 1/2J_CH_. Alternatively, in this work, data for the upfield and downfield lactate peaks were acquired sequentially using a narrowband SPSP RF pulse. The lactate distribution appeared similarly across images of downfield and upfield signals prior to summation as seen from high correlation and SSIM values, and artifacts arising from J_CH_-coupled signals were not observed. The whole brain *k*_PL_ values obtained using the summed lactate signal are similar to those reported for [1–^13^C]lactate in the healthy human brain, indicating that the peak splitting and *T*_1_ differences can be properly accounted for in the kinetic model (Autry et al., 2020). The large chemical shift separation between [2–^13^C]pyruvate and [2–^13^C]lactate may also be relevant at lower fields where *T*_1_ relaxation times are shorter.

## 5. Conclusions

This research project developed a new approach using a specialized HP ^13^C MR RF pulse sequence for acquiring volumetric and dynamic EPI of HP [2–^13^C]pyruvate metabolism to [5–^13^C]glutamate and to [2–^13^C] lactate probing glycolytic and oxidative metabolism simultaneously, and demonstrated feasibility and initial results in five normal volunteer studies. The strategy of separately exciting the two peaks of the lactate doublet was shown to be feasible for imaging this metabolite with signal splitting. In combination with a variable resolution approach, the HP metabolite signals were utilized for quantifying *k*_PL_ and *k*_PG_ throughout the brain of healthy volunteers providing new measures of cerebral energy metabolism in humans enabling transition from prior research in the rat brain (Park et al., 2013; Park et al., 2016).

This study quantified the metabolism of HP [2–^13^C]pyruvate to [2–^13^C]lactate and [5–^13^C]glutamate throughout the healthy human brain for the first time. Measuring of spatial localizations of pyruvate-to-lactate and pyruvate-to-glutamate conversions signifies an important step to use HP [2–^13^C]pyruvate to investigate cerebral energy metabolism and potentially characterize brain tumors with isocitrate dehydrogenase (IDH) mutations.

## Supplementary Material

Supplementary Material

## Data Availability

Data will be made available on request.
