# Supplementary material for: Hyperpolarized [2–13C]pyruvate MR molecular imaging with whole brain coverage
Source: Neuroimage. Author manuscript; Available in PMC 2024 Oct 15. (PMC10530049; doi:10.1016/j.neuroimage.2023.120350)
Supplement: Supplementary Material [file NIHMS1931960-supplement-Supplementary_Material.pdf]

# Supplementary Material for "Hyperpolarized [2-$^{13}$C]Pyruvate MR Molecular Imaging with Whole Brain Coverage"

Brian T. Chung*[1,2], Yaewon Kim*[1], Jeremy W. Gordon[1], Hsin-Yu Chen[1], Adam W. Autry[1], Philip M. Lee[1,2], Jasmine Y. Hu[1,2], Chou T. Tan[3], Chris Suszczynski[3], Susan M. Chang[4], Javier E. Villaneuva-Meyer[1], Robert A. Bok[1], Peder E.Z. Larson[1,2], Duan Xu[1,2], Yan Li[1], Daniel B. Vigneron[1,2,4]

[1]Department of Radiology and Biomedical Imaging, University of California, San Francisco, CA 94158, USA
[2]UCSF – UC Berkeley Graduate Program in Bioengineering, University of California, USA
[3]ISOTEC Stable Isotope Division, MilliporeSigma, Merck KGaA, Miamisburg, OH 45342, USA
[4]Department of Neurological Surgery, University of California, San Francisco, CA 94158, USA

# Supplementary Figures

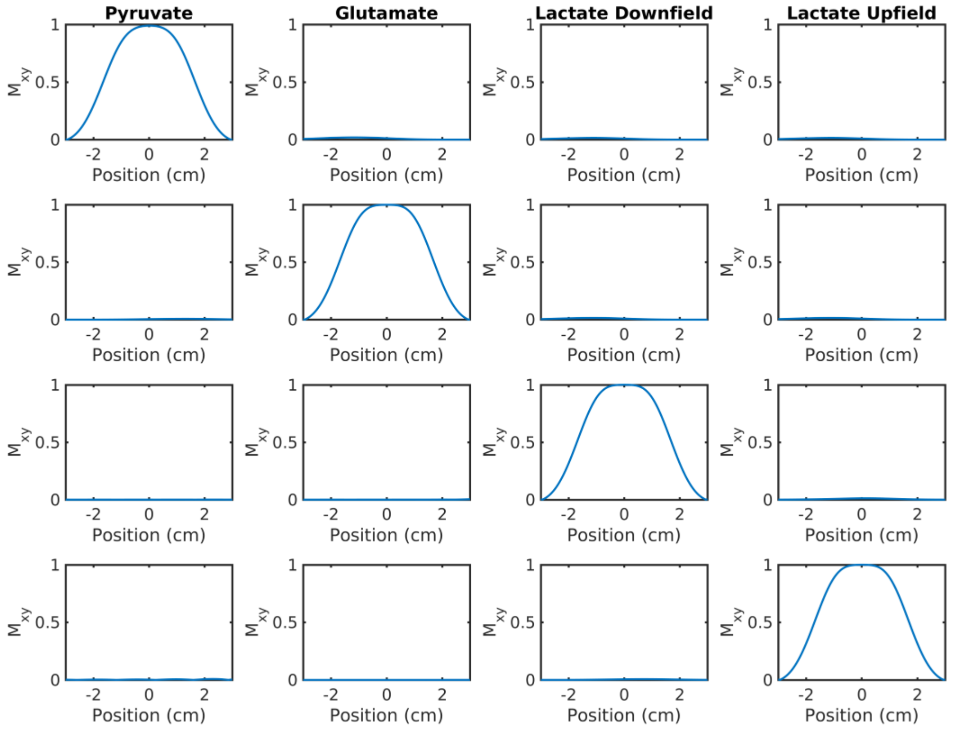


Supplementary Figure 1: Simulation of on- and off-resonance responses for four frequencies corresponding to metabolites of interest (Pyruvate = 0 Hz; Glutamate = -771 Hz; Lactate downfield = -4294 Hz; Lactate upfield = -4437 Hz). SPSP RF pulse specifications are: 18.1ms pulse width, 114 Hz passband (full-width at half-maximum), and 489 Hz between replicating passbands.

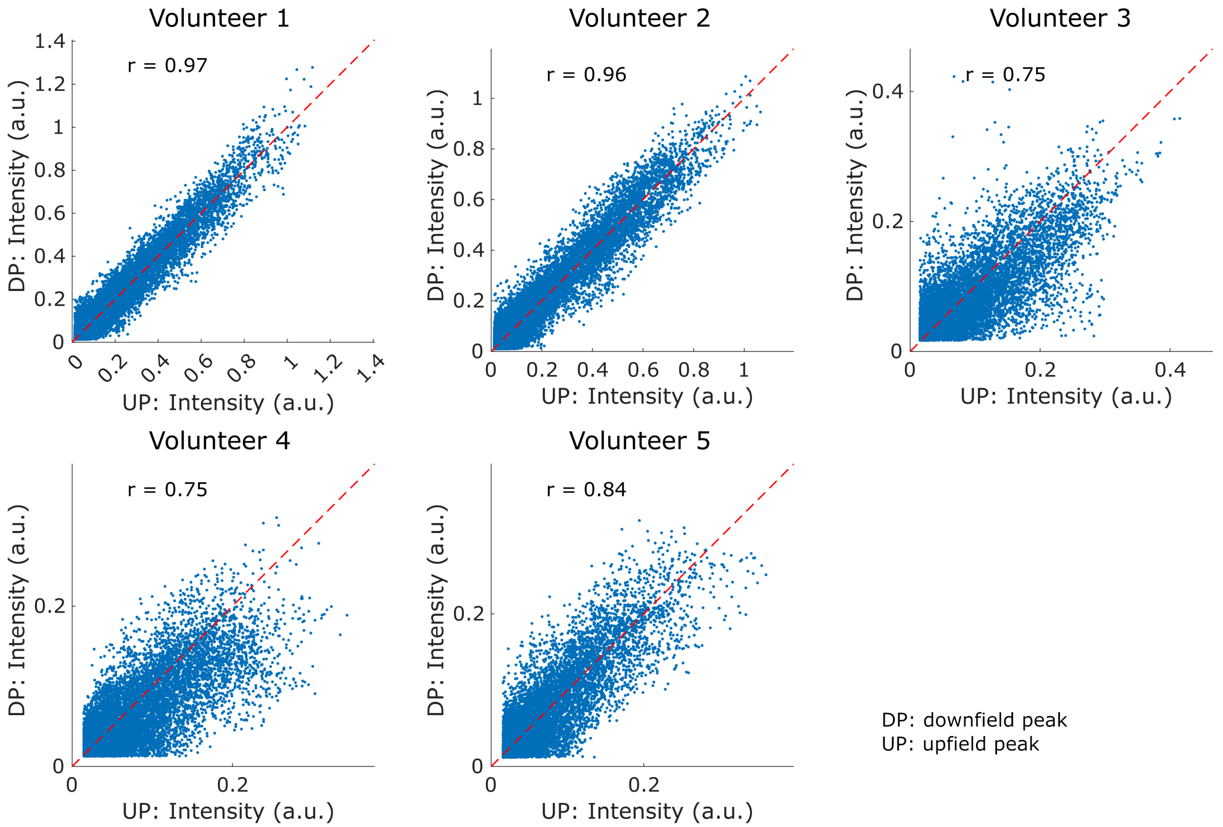


Supplementary Figure 2: Comparison of signal intensities between upfield and downfield peaks of the [2-$^{13}$C]lactate doublet signals, obtained from the healthy volunteer data (n = 5). The comparison excluded signals with SNR lower than 3. The red diagonal indicates 1:1 agreement between the downfield and upfield lactate peaks (DP and UP, respectively). The Pearson correlation (r) values are included in each plot.

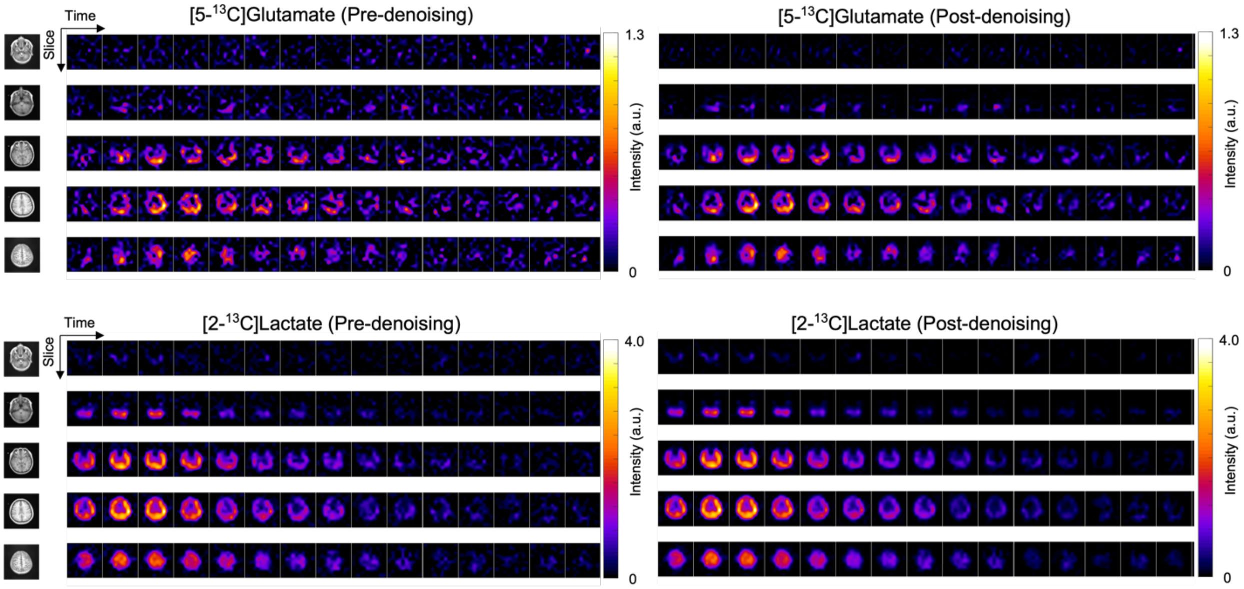


Supplementary Figure 3: Dynamics of HP [5-$^{13}$C]glutamate and [2-$^{13}$C]lactate (downfield peak) throughout the human brain of a healthy volunteer (Vol-1). Displayed images show the first 15 timeframes with 3 second temporal resolution before (left column) and after (right column) denoising using a patch-based HOSVD method[1]. Shown on the left are $^{1}$H IR-SPGR anatomy images capturing average of slices.

Reference:

1. Kim Y, Chen HY, Autry AW, Villanueva-Meyer J, Chang SM, Li Y, Larson PEZ, Brender JR, Krishna MC, Xu D, Vigneron DB, Gordon JW. Denoising of hyperpolarized $^{13}$C MR images of the human brain using patch-based higher-order singular value decomposition. Ma*gn Reson Med*. 2021;86(5):2497-2511. doi:10.1002/mrm.28887
